# Ultrasound-guided five-point injection of botulinum toxin for patients with trapezius hypertrophy

**DOI:** 10.1186/s13018-021-02758-y

**Published:** 2021-10-22

**Authors:** Wanying Chen, Xiaoyu Zhang, Yingying Xu, Zemin Xu, Haiyan Qin, Lianbo Zhang

**Affiliations:** 1 Department of Plastic Surgery, The Third Hospital of Jilin University, No.126, Xiantai Street, Erdao District, Changchun, 130000 Jilin China; 2 Department of Gastrointestinal and Colorectal Surgery, The Third Hospital of Jilin University, Changchun, 130000 Jilin China; 3 Department of Ultrasound, The Third Hospital of Jilin University, Changchun, 130000 Jilin China; 4 Orthopedics Department, The Third Hospital of Jilin University, Changchun, 130000 Jilin China

**Keywords:** Trapezius hypertrophy, Five-point, Botulinum toxin type A

## Abstract

**Objectives:**

Our study aimed to explore the clinical therapeutic effects of ultrasound-guided five-point injection of botulinum toxin type A for patients with trapezius hypertrophy.

**Methods:**

Twenty female patients diagnosed with trapezius hypertrophy were enrolled in this study. The thicknesses of the trapezius muscle were measured by using the ultrasound scanner to locate the thickest point of trapezius, followed by labelling the other four points around the first point. Botulinum toxin type A was injected bilaterally (50 IU/side, 5 points/side) in the trapezius muscle of these patients. The surgery effects were evaluated by thicknesses of the trapezius muscle, intramuscular needle electromyographic and electroneurographic examinations, appearance changes and patients’ satisfactions.

**Results:**

Statistically significant differences in thicknesses of the trapezius muscle were observed at 4 weeks (*p* < 0.001), 12 weeks (*p* < 0.001), 20 weeks (*p* < 0.001), 28 weeks (*p* = 0.011), 36 weeks (*p* = 0.022), and 44 weeks (*p* = 0.032) after surgery. The latencies of trapezius muscle became longer at 12 weeks after surgery (left: 2.40 ms, right: 2.53 ms vs. left: 1.75 ms, right: 2.00 ms). Electroneurographic results showed amplitude reduction of compound muscle action potentials (CMAPs) at 12 weeks after surgery (left: 1.91 uV, right: 3.10 uV vs. left: 15.00 uV, right: 15.40 uV). Obvious appearance changes were revealed at 12 weeks after surgery. All of 80% patients were very satisfied, 15% patients were relatively satisfied, and 5% patients were not satisfied with the surgery.

**Conclusion:**

Ultrasound-guided five-point injection of botulinum toxin type A might be effective for patients with trapezius hypertrophy.

## Highlights

1. Twenty female patients with trapezius hypertrophy were included in this study.
2. The thickest point of trapezius was located by ultrasound scanner to label the other four points for BOTOX injection.
3. It showed 80% patients were very satisfied, 15% relatively satisfied, and 5% not satisfied with the surgery.

## Introduction

Human trapezius is a triangular muscle with the superior muscle fibers attached to the posterior aspect of the neck and the inferior fibers attached to the base of the spine of the scapula [1]. The muscular tension and hypertrophy of trapezius muscle predominantly affect the female population and could be caused by strain, pain, psychological stress, and myofascial tender- and trigger-points, leading to myalgia, primary fibromyalgia, and craniomandibular disorders [2, 3]. Therefore, it is really important to find effective treatments for patients with trapezius hypertrophy.

The anaerobic bacteria, *Clostridium botulinum*, produce a family of seven serologically distinguishable neurotoxins (type A, B, C1, D, E, F, and G), and each neurotoxin cleaves a different intracellular protein or the same target at distinct bonds [4]. The botulinum toxin type A is best known because of its successful and widespread clinical use for many disorders, such as headache (myofascial pain syndromes, migraine, and other headache types) [5, 6], spasticity [7], and cranial-cervical dystonia [8]. Botulinum toxin type A can selectively cleave synaptosomal protein with a molecular weight of 25 kDa (SNAP-25) and inhibit the release of the neurotransmitter, acetylcholine, at the neuromuscular junction thereby inhibiting striated muscle contractions [9, 10]. Thus, the botulinum toxin type A treatment often reduces the pain associated with headache, cervical dystonia and achalasia. However, few studies have reported about the botulinum toxin type A injection treatment methods for trapezius hypertrophy. The effects of botulinum neurotoxin are dependent on the location, concentration, and volume of the solution injected [11, 12]. Thus, it is necessary to standardize the injection procedures to improve the therapeutic effects, especially the locating method for injection sites. In the study of Zhou et al. [13], 30 women with bilateral trapezius hypertrophy were treated with botulinum toxin type A injection. The injection points were selected according to the following instructions: the intersection point between the lateral clavicle margin and the superior edge of the trapezius muscle is set as ‘A’ point; the intersection point between the vertical line of neck and the superior edge of the trapezius muscle is set as ‘H’ point; a line connecting the points ‘A’ and ‘H’ is drawn at the top edge of the trapezius in frontal view; ‘G’ point is marked from point A to the inside of the AH arc with the direct distance of 2 cm; taking H point as a perpendicular line and G point as a horizontal line, the range of two lines and GH arc is the injection area where located about 5–9 consecutive injection sites with interval of 1–2 cm per side. However, some patients showed no obvious improvement. Therefore, it is still necessary to develop more effective ways for injection of botulinum toxin for treating patients with trapezius hypertrophy.

Our study enrolled twenty female patients with trapezius hypertrophy. They underwent the ultrasound-guided five-point injection of botulinum toxin type A. Briefly, the thicknesses of the trapezius muscle were measured by using the ultrasound scanner to locate the thickest point of trapezius, followed by labelling the other four points around the first point with the distance of 2 cm. In order to deliver drug appropriately, the botulinum toxin type A injections were carried out under the guidance of ultrasound scanner. Botulinum toxin type A was injected bilaterally in the trapezius muscle of these labelled sites. The long-term effects of botulinum neurotoxin A injected in muscle might be the local changes in the muscle fiber size, electromyographic abnormalities, and reversible denervation atrophy [14]. After surgery, thicknesses of the trapezius muscle were measured, intramuscular needle electromyographic and electroneurographic examinations were carried out, appearance changes and patients’ satisfactions were recorded.

## Materials and methods

### Population

Twenty female patients meeting the inclusion and exclusion criteria were recruited in outpatient clinics of plastic surgery department at China-Japan Union Hospital of Jilin University from January 2015 to January 2018. All procedures in our study were approved by the institutional ethics committee, and the enrolled patients had been given written informed consents.

Inclusion and exclusion criteria were as follows: female patients diagnosed with bilateral trapezius hypertrophy feeling muscle tension and soreness; age between 20 and 50 years; healthy liver and kidney function; no shoulder lesion; no sign of coagulation dysfunction, myasthenia gravis, neurological disorders or other major diseases; not taking blood-activating drugs (such as aspirin) one week before surgery; no history of receiving specific training and other treatments within one year; no plan of pregnancy in the next year; had willingness and ability to comply with study procedures. Before the surgery, two attending physicians and one chief physician in the plastic surgery department participated in the evaluations of muscular tension based on the Ashworth Scale [15], physical performance based on the Fugl–Meyer Scale [16] and activities of daily living for patients based on the Barthel Index [17]. Patients with good muscular tension, motor functions and activities of daily living were included.

### Surgery treatments

**Fig. 1:**
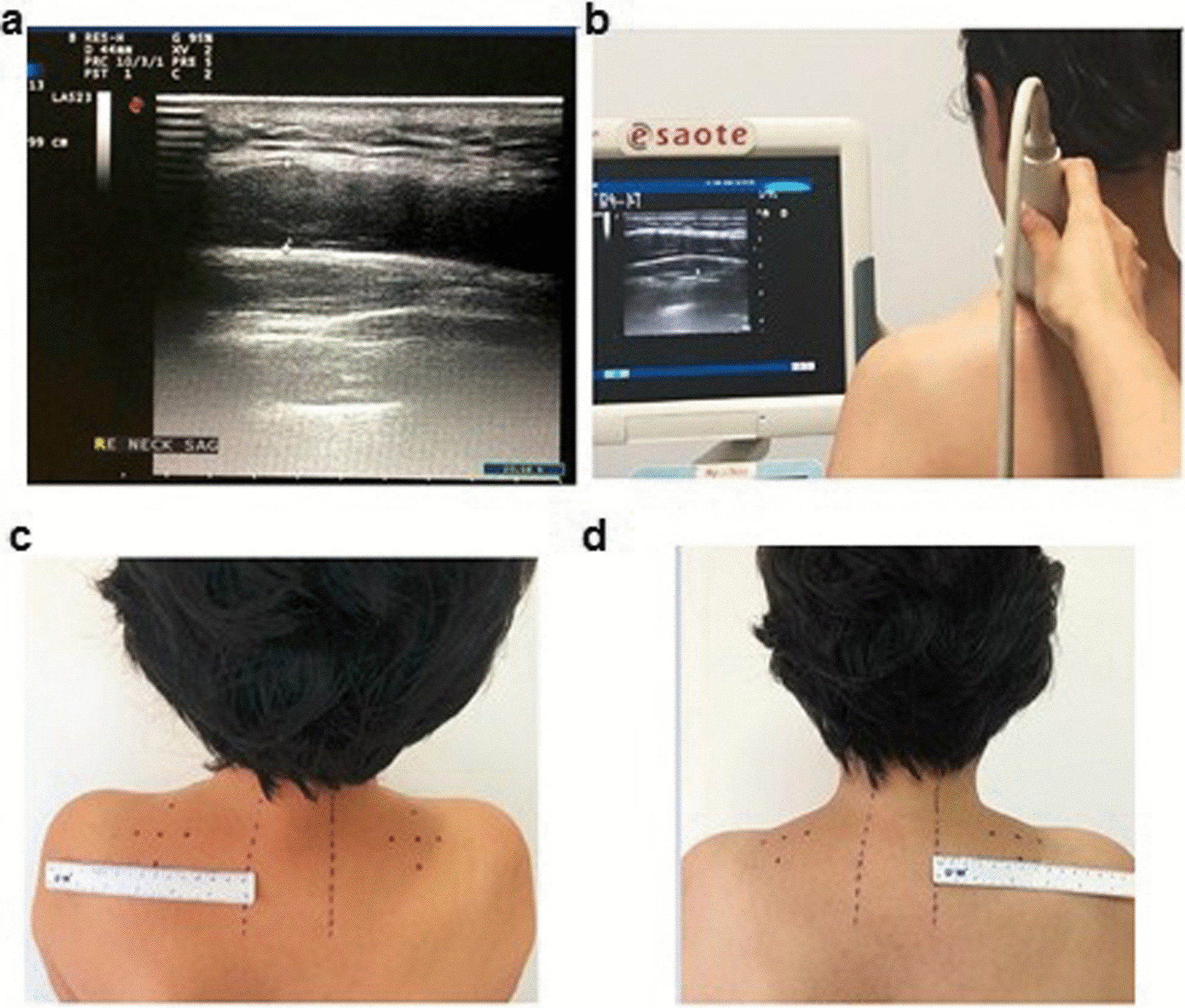
Measurement of muscle thickness and labeling of botulinum toxin type A (BOTOX) injected points. **a**, **b** The muscle thickness was measured by using the ultrasound scanner Esaote MyLab Twice; **c** the five points at left trapezius muscle for BOTOX administration; **d** the five points at right trapezius muscle for BOTOX administration

The botulinum toxin type A (BOTOX, Allergan Corp, Irvine, CA; Lot C3611C3; 100 IU/tube) was dissolved in normal saline (3 mL per tube) for administration. Measurements of muscle thickness were made by using the ultrasound scanner Esaote MyLab Twice (Esaote SpA, Genova, Italy) to locate the thickest point of each side trapezius muscle for every patient who adopted a sitting position with their back to the doctor (Fig. 1a, b). The thickest point was set as the center to select and label the other four points (up, down, left and right) with the distance of 2 cm (Fig. 1c, d). Patients received botulinum toxin type A (BOTOX) injection bilaterally (0.3 mL/point, 5 point/side; 100 IU totally) with the speed of 0.05 mL/s using a 1-mL syringe at the labeled points on trapezius muscle. The injection procedure was done directly since it is acceptable for all the patients. In order to deliver BOTOX appropriately, the BOTOX injections were carried out under the guidance of ultrasound scanner on both sides. Before giving botulinum toxin A, injection procedure should not be scheduled during the menstrual period. After giving botulinum toxin A, patients should avoid rinsing skin with water on that day or taking blood-activating drugs.

### Assessments and follow-up visits

The thicknesses of the trapezius muscle were measured by using the ultrasound scanner at 4, 12, 20, 28, 36, and 44 weeks after surgery. The intramuscular needle electromyographic examination was performed using a Dantec Keypoint electromyograph (Dantec, Keypoint, Medtronic; type 9031A070). The thickest point of each side trapezius muscle was located to assess the spontaneous electrical activity in a resting state and recruiting response at motion state. Electroneurographic evaluations of compound muscle action potential (CMAP) latency (LAT) and amplitude (AMP) were performed.

All the patients were followed up for 1 year to record the thickness of trapezius muscle and collect corresponding appearance photos. Patients’ satisfaction with shoulder appearance and muscle comfortability was investigated through questionnaire method.

### Statistical analysis

All measurement data were expressed as mean ± standard deviation (SD). Paired *t*-test was adopted to analyze the differences between two groups. All the statistical analyses were conducted using the SPSS v22.0 software (Chicago, IL, USA), and *p* < 0.05 was considered to be statistically significant.

## Results

### Thickness of trapezius muscle

**Fig. 2:**
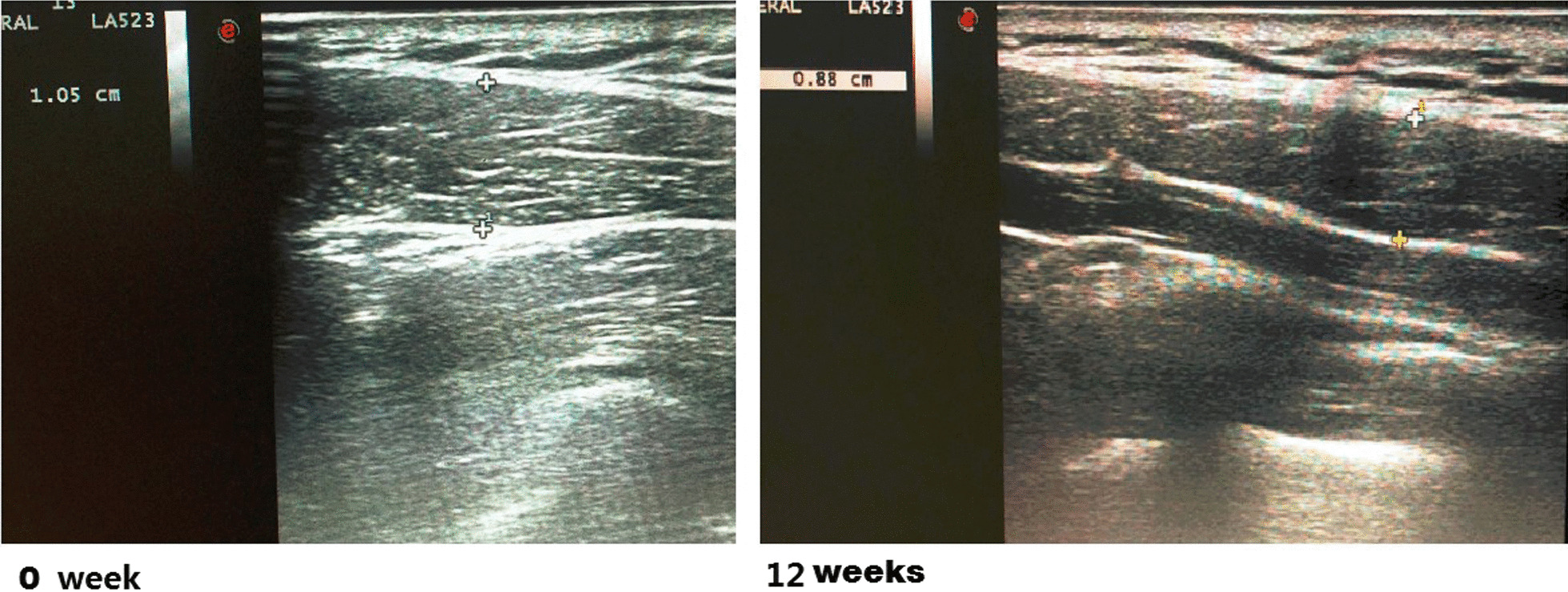
The muscle thicknesses before and after surgery were measured by using the ultrasound scanner

The thicknesses of trapezius muscle on both sides for each patients before (0 week) and after surgery were measured (Fig. 2; Table 1). Statistically significant differences were observed at 4 weeks (*p* = 1.9150E−20, *t* = 17.9436), 12 weeks (*p* = 3.4143E−20, *t* = 17.6481), 20 weeks (*p* = 1.7523E−17, *t* = 14.6892), 28 weeks (*p* = 1.2023E−15, *t* = 12.8968), 36 weeks (*p* = 4.8321E−16, *t* = 13.2702), and 44 weeks (*p* = 2.9239E−15, *t* = 12.5392) after surgery when compared with preoperatively.

**Table 1:** The thicknesses of trapezius muscle on the left and right sides before surgery and after surgery according to the ultrasound scanner

Patient	Before surgery		4 weeks		12 weeks		20 weeks		28 weeks		36 weeks		44 weeks	
No.	Left	Right	Left	Right	Left	Right	Left	Right	Left	Right	Left	Right	Left	Right
1	1.39	1.31	1.26	1.24	1.19	1.15	1.26	1.21	1.28	1.23	1.29	1.25	1.31	1.26
2	1.13	1.22	1.02	1.10	0.96	1.04	1.05	1.10	1.07	1.14	1.08	1.15	1.09	1.16
3	1.32	1.42	1.25	1.35	1.23	1.33	1.29	1.38	1.29	1.38	1.30	1.39	1.31	1.40
4	1.26	1.29	1.13	1.10	1.13	1.11	1.17	1.18	1.20	1.21	1.20	1.21	1.20	1.21
5	0.99	1.10	0.90	0.98	0.86	0.97	0.90	1.01	0.92	1.02	0.94	1.03	0.95	1.04
6	1.10	1.21	1.05	1.15	0.99	1.10	1.02	1.11	1.04	1.15	1.06	1.15	1.06	1.15
7	1.25	1.32	1.13	1.19	1.09	1.13	1.11	1.16	1.16	1.21	1.18	1.25	1.19	1.25
8	1.24	1.20	1.03	1.00	1.01	0.99	1.09	1.06	1.16	1.13	1.17	1.14	1.17	1.15
9	1.23	1.33	1.10	1.19	1.06	1.11	1.09	1.16	1.12	1.18	1.14	1.19	1.16	1.21
10	1.55	1.52	1.32	1.29	1.34	1.31	1.41	1.40	1.41	1.40	1.41	1.40	1.40	1.39
11	1.22	1.10	1.07	0.98	1.06	1.00	1.10	1.02	1.10	1.03	1.11	1.03	1.12	1.03
12	1.44	1.30	1.21	1.16	1.10	1.05	1.25	1.19	1.34	1.26	1.36	1.26	1.36	1.26
13	1.25	1.28	1.12	1.10	1.14	1.12	1.21	1.23	1.21	1.23	1.21	1.23	1.21	1.23
14	1.39	1.35	1.31	1.25	1.33	1.26	1.34	1.27	1.36	1.30	1.36	1.30	1.36	1.29
15	1.05	1.01	0.89	0.83	0.88	0.80	0.90	0.87	0.93	0.91	0.93	0.90	0.93	0.91
16	1.24	1.10	1.02	0.93	0.95	0.87	1.03	1.00	1.15	1.03	1.15	1.04	1.15	1.04
17	1.03	1.06	0.85	0.88	0.87	0.89	0.98	1.01	0.99	1.02	0.99	1.02	0.98	1.02
18	1.34	1.32	1.22	1.17	1.14	1.11	1.16	1.13	1.18	1.15	1.21	1.20	1.23	1.21
19	1.17	1.10	1.05	0.95	1.07	1.00	1.11	1.03	1.12	1.07	1.13	1.07	1.14	1.07
20	1.14	1.11	1.04	1.01	1.02	1.00	1.06	1.03	1.10	1.09	1.09	1.08	1.10	1.09

**Fig. 3:**
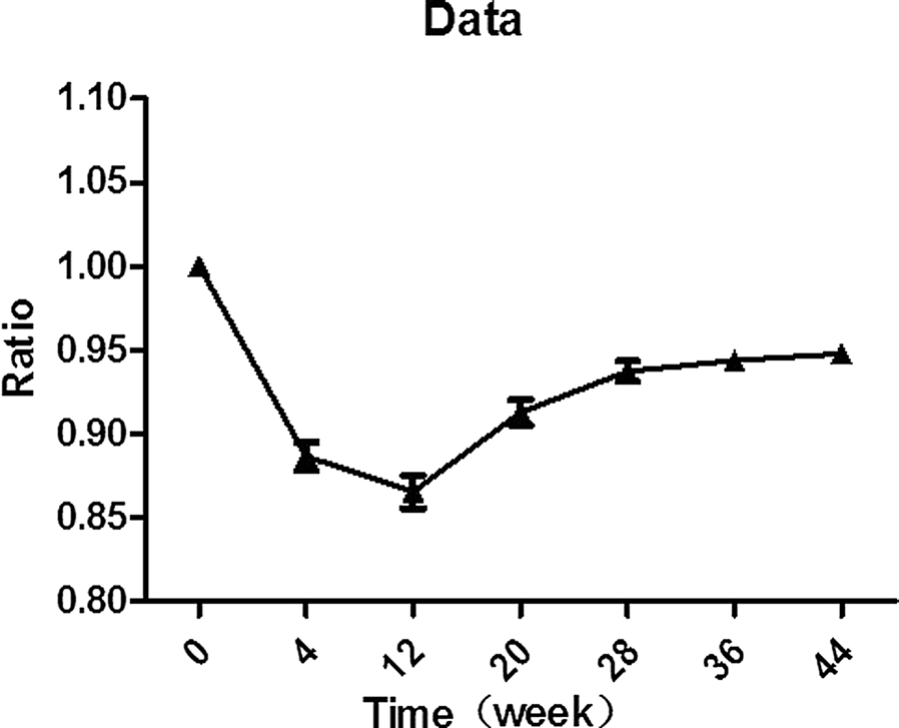
The relative ratios of thicknesses of trapezius muscle at 4 weeks, 12 weeks, 20 weeks, 28 weeks, 36 weeks, and 44 weeks after surgery compared with those before surgery

In order to reflect the change tendency more clearly, the thicknesses of trapezius muscle after surgery were compared with those before the surgery to calculate the relative ratios. The relative ratios at 4 weeks, 12 weeks, 20 weeks, 28 weeks, 36 weeks, and 44 weeks after surgery were 0.887 ± 0.038, 0.866 ± 0.044, 0.913 ± 0.034, 0.937 ± 0.028, 0.944 ± 0.024, and 0.948 ± 0.024, respectively (Fig. 3).

### Intramuscular needle electromyographic and electroneurographic examinations

**Fig. 4:**
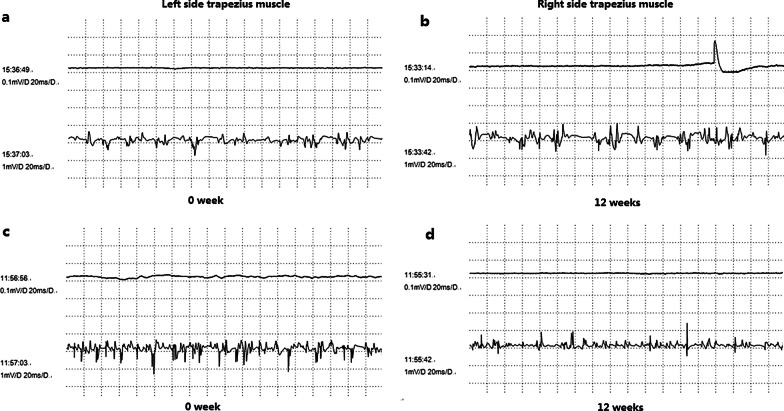
Electromyographic measurements of left and right trapezius muscles before surgery (0 week) and at 12 weeks after surgery

Before BOTOX injection (0 week), all patients showed normal electromyographic patterns of left and right trapezius muscle (Fig. 4a, b). No spontaneous electrical activity was observed in a resting state before surgery. At 12 weeks after the surgery, spontaneous electrical activity in a resting state, low-amplitude and polyphasic motor unit potentials (MUPs) were captured during slight force at motion state. Under the maximum force, polyphasic potentials with shorter duration and attenuated amplitude were detected. The latencies of trapezius muscle before surgery (left: 1.75 ms; right: 2.00 ms) were shorter than those at 12 weeks after surgery (left: 2.40 ms; right: 2.53 ms). The amplitudes of CMAPs were reduced at 12 weeks after surgery (left: 1.91 uV; right: 3.10 uV) when compared with before surgery (left: 15.00 uV; right: 15.40 uV) (Fig. 4c, d).

### Appearance and satisfaction

**Fig. 5:**
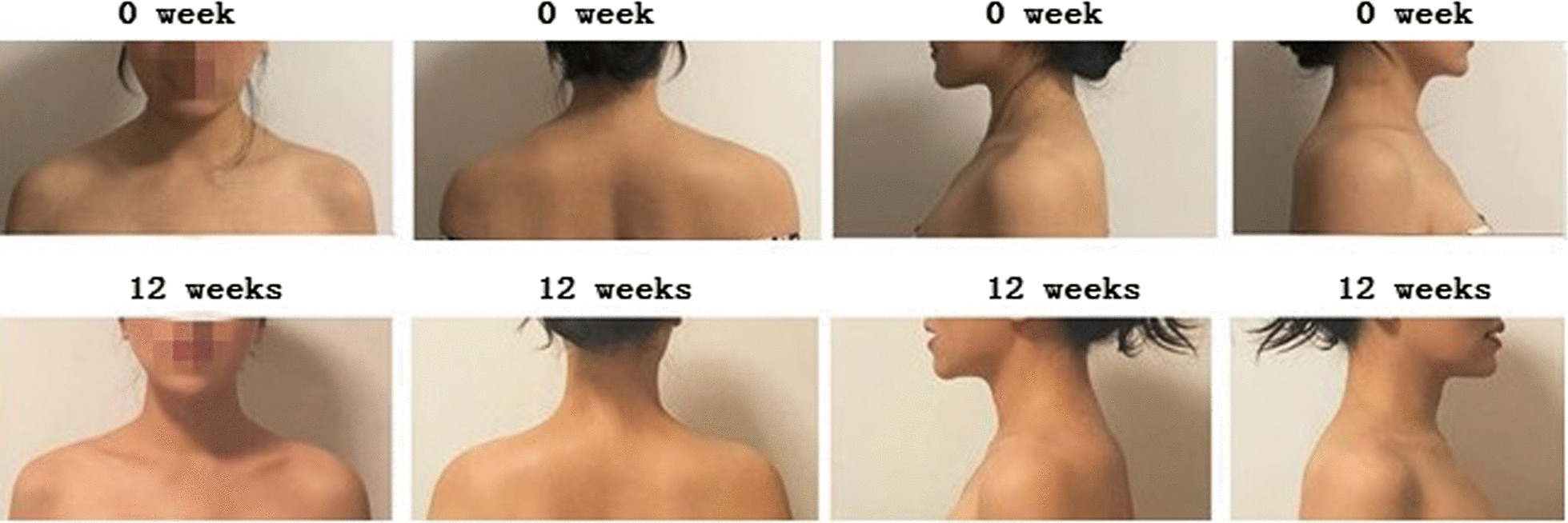
Changes in appearance before surgery (0 week) and at 12 weeks after surgery

The appearances were obviously different at 12 weeks after surgery from before surgery (Fig. 5). According to the questionnaires, 80% (16/20) patients were very satisfied with the surgery, 15% (3/20) patients were relatively satisfied with the treatment results, and 5% (1/20) patients were not satisfied with the surgery. Meanwhile, patients were most satisfied with the results at the first and third months after treatment. Muscle weakness was observed in one subject within the first month, and this side effect was eliminated after 1 month.

## Discussion

Nerves could exert their influence on extrasynaptic receptor density either directly, via a chemical messenger, or indirectly, through the activity evoked by synaptic transmission. Synaptic transmission is process by which signals are transferred from a neuron to its target which is a fundamental function of neurons [18]. At the neuromuscular junction, the presynaptic nerve ending terminals are filled with 50 nm diameter synaptic vesicles containing the neurotransmitter, acetylcholine, and many vesicles are clustered at dense patches (active zones) on the cytoplasmic side of that part of the surface membrane facing the muscle fibers [19, 20]. The mouths of junctional folds of the postsynaptic muscle membrane containing acetylcholine receptors are precisely aligned with the presynaptic active zones [19]. Synaptic vesicles fuse with the membrane in response to an elevation of intraneuronal calcium concentration that results in a rapid increase in exocytosis of the neurotransmitter [21]. The intracellular proteins including SNAP-25, vesicle-associated membrane protein (VAMP, also known as synaptobrevin), and syntaxin could contribute to the fusion of the vesicles with the plasma membrane during exocytosis [22]. Botulinum toxin type A has proteolytic actions on separate cleavage sites in SNAP-25 contribute to inhibiting the release of acetylcholine that result in muscle relaxation, thereby long used in the successful treatments of various dystonias [22].

Although botulinum toxin A injections are one of the most recent and popular methods for inducing muscle atrophy, the injection dose and operation procedures are still need to be studied for maintenance of a long-lasting effects on muscular hypertrophy [23]. Previous studies showed that three-dimensional computerized tomographic (3D-CT) scanning or magnetic resonance imaging (MRI) of the whole head was performed before the administration of BoNTA to make the injection site for botulinum toxin A [24, 25]. However, there are still some problems with 3D-CT and MRI, such as radiation and costs, which caused difficulties in data collections and long-term follow-up visits for large sample size. Handa et al. revealed that ultrasonic duplex Doppler method is non-invasive, accurate for screening tests available for renovascular hypertension and quite useful for follow-up examinations in the patients with angioplasty [26]. Meanwhile, it has been proved that masseter muscle thickness could be accurately measured by ultrasonography [27]. B mode, Doppler and ultrasound elastography imaging has been used for locating active trigger point for dry needling in treatment of female patients with myofascial pain syndrome [28]. It has been demonstrated real-time ultrasonography could even be used for non-symptomatic females, with regarding to the measurement of morphometric features of upper trapezius, its stiffness and blood supply, which could provide numerical reference value and objective assessment for clinical plans [29]. Therefore, we adopted the ultrasound scanner to measure the thicknesses of the trapezius muscle for all the enrolled twenty female patients to accurately locate the thickest point of trapezius, followed by labelling the other four points around the first point. Botulinum toxin type A was injected bilaterally in the trapezius muscle of these labelled sites.

The relative ratios of trapezius muscle thickness at 4 weeks, 12 weeks, 20 weeks, 28 weeks, 36 weeks, and 44 weeks after surgery were 0.887 ± 0.038, 0.866 ± 0.044, 0.913 ± 0.034, 0.937 ± 0.028, 0.944 ± 0.024, and 0.948 ± 0.024, respectively. The period of 4–12 weeks after injection had the most obvious reduction in trapezius muscle thickness. In a retrospective study of 1021 clinical patients treated with botulinum toxin type A for remodeling the lower facial contour line, the thickness of the muscle was reduced by 31% according to ultrasonograms three months after the botulinum toxin A injection with the maximum effect showing after 10 to 12 weeks [30]. The effects of fatigue on joint position sense error in subclinical myofascial pain syndrome participants could be evaluated by surface electromyography [31]. The global pattern of muscle recruitment was consistent between surface electromyography and intramuscular fine-wire electromyographic, but surface electromyography recordings were characterized by additional myoelectric content [32]. Thus, in our study, records of intramuscular needle electromyographic and electroneurographic examinations before and after injection have been used to document changes in muscle function. Before BOTOX injection, all patients showed normal electromyographic patterns of left and right trapezius muscle, and no spontaneous electrical activity was observed in a resting state. At 12 weeks after the injection, spontaneous electrical activity in a resting state, low-amplitude and polyphasic motor unit potentials were captured during slight force at motion state. Under the maximum force, polyphasic potentials with shorter duration and attenuated amplitude were detected. In the study of Lee et al., the maximal amplitude of the right and left masseter muscles decreased to the lowest value one month after botulinum toxin A injections, with continuous increase being observed thereafter [33]. Kim et al. showed there was a 43.8% decrease in the electromyographic values and 28.7% decrease in the 35-U group at 24 weeks after injection [34]. In our study, the latencies of trapezius muscle at 12 weeks after surgery became longer and the amplitudes of CMAPs were reduced. The appearances were obviously different at 12 weeks after surgery from before surgery. Meanwhile, 80% patients were very satisfied, 15% patients relatively satisfied, and 5% patients were not satisfied due to muscle weakness. Meanwhile, patients were most satisfied with the results at the first and third months after treatment. However, it is still unclear whether maximum effects were obtained at 12 weeks after injection. There are still some limitations in our study. One of the limitations in our study was that only twenty female patients meeting the inclusion and exclusion criteria were recruited. The treatment efficacy of ultrasound-guided five-point injection of botulinum toxin type A for patients with trapezius hypertrophy still needs to be validated in a larger cohort of patients in the future. Another limitation of our study was that the therapeutic effect of botulinum toxin type A on trapezius hypertrophy was reduced after 20–28 weeks, and whether the treatment efficacy could be strengthened by increasing the injection dose was not studied. Thus, it is also necessary to explore the relationship between botulinum toxin dose and the thickness of trapezius muscle in the future study.

## Conclusions

In conclusion, the ultrasound-guided five-point injection of botulinum toxin type A was effective in treating trapezius hypertrophy. However, further studies with longer tracking period or additional injections are required to better evaluate the treatment effects, and the appropriate timing for additional injections.

## Data Availability

Not applicable.

## Abbreviations

CMAP: Compound muscle action potential
LAT: Latency
AMP: Amplitude
SD: Standard deviation
MUPs: Motor unit potentials
MRI: Magnetic resonance imaging
